# Effect of UV Radiation on Optical Properties and Hardness of Transparent Wood

**DOI:** 10.3390/polym13132067

**Published:** 2021-06-23

**Authors:** Igor Wachter, Tomáš Štefko, Peter Rantuch, Jozef Martinka, Alica Pastierová

**Affiliations:** Department of Integrated Safety, Faculty of Materials Science and Technology in Trnava, Slovak University of Technology in Bratislava, Botanická 49, 917 24 Trnava, Slovakia; tomas.stefko@stuba.sk (T.Š.); peter.rantuch@stuba.sk (P.R.); jozef.martinka@stuba.sk (J.M.); alica.pastierova@stuba.sk (A.P.)

**Keywords:** transparent wood, UV-C radiation, optical properties, basswood, hardness, chromophores deactivation

## Abstract

Optically transparent wood is a type of composite material, combining wood as a renewable resource with the optical and mechanical properties of synthetic polymers. During this study, the effect of monochromatic UV-C (λ—250 nm) radiation on transparent wood was evaluated. Samples of basswood were treated using a lignin modification method, to preserve most of the lignin, and subsequently impregnated with refractive-index-matched types of acrylic polymers (methyl methacrylate, 2-hydroxyethyl methacrylate). Optical (transmittance, colour) and mechanical (shore D hardness) properties were measured to describe the degradation process over 35 days. The transmittance of the samples was significantly decreased during the first seven days (12% EMA, 15% MMA). The average lightness of both materials decreased by 10% (EMA) and 17% (MMA), and the colour shifted towards a red and yellow area of CIE L*a*b* space coordinates. The influence of UV-C radiation on the hardness of the samples was statistically insignificant (W+MMA 84.98 ± 2.05; W+EMA 84.89 ± 2.46), therefore the hardness mainly depends on the hardness of used acrylic polymer. The obtained results can be used to assess the effect of disinfection of transparent wood surfaces with UV-C radiation (e.g., due to inactivation of SARS-CoV-2 virus) on the change of its aesthetic and mechanical properties.

## 1. Introduction

Wood, as a renewable and earth-abundant resource, is a well-established material in many applications due to its good physical and chemical properties, including high strength, low thermal conductivity, non-toxicity, and biodegradability [1,2]. The future of sustainable development depends on how humans will transfer their dependability from finite fossil-based materials to sustainable and renewable materials to combat the climate change. Recently, there is an increasing number of articles dealing with eco-friendly composites [3,4].

Wood composite materials are engineered and produced with tailored physical and mechanical properties appropriate for a wide variety of applications, both known and not discovered yet [5].

Transparent wood is a composite material consisting of a modified wood component (deactivated chromophores or delignification) and an in situ polymerized, transparent component. Transparent wood has received much attention, owing to its great potential for applications in light-transmitting buildings, which can partially replace artificial light with sunlight and therefore save energy [6,7]. Transparent wood can be used to produce building [8,9], solar cells [10] and magnetic materials [11]. Additional functionalization has been demonstrated, such as lasing [12], heat shielding [13], thermal energy storage [14], electroluminescent devices [15], and combined with conducting polymers in electromechanical devices [16].

To fabricate transparent wood, two steps are typically involved: completely removing the light-absorbing lignin from the cell walls of natural wood by a solution-based immersion method and infiltrating a refractive index matching polymer into the delignified wood matrix to minimize light absorption and scattering, respectively [9,17,18,19,20].

Alternatively, many studies have focused on physically and/or chemically modifying lignin structures to reduce lignin colour and impart new functionalities, including fractionation [21], grind [22], acetylated lignin [23,24], fragmented lignin [25,26] and metal-decorated lignin [27,28].

Although considered critical in previous publications, delignification processes are time consuming and not necessarily environmentally friendly because of the production of odorous components and chlorinated compounds. Moreover, the removed lignin significantly weakens the wood structure so it can be challenging to work with such a fragile material, and this also lowers the number of suitable wood species for transparent wood preparation; pine and spruce, for example, breaks into pieces after the delignification step. The lignin modification method is superior to the delignification process in the following four aspects [29]:

(1) The lignin modification method is completed in a short time.
(2) Lignin is largely retained, and the wood structure is therefore better preserved.
(3) Lignin-modified wood templates show better mechanical properties.
(4) The lignin modification method is a green process since toxic effluents are minimized.

Also, it is important to note that wood consists of around 30 wt% of lignin which provides structural support and therefore the transparent wood fabricated by this process could be considered more environmentally friendly because less synthetic polymer is needed for its fabrication.

There is an increasing number of studies with various applications for the use of transparent wood where it needs to withstand outdoor weather conditions from which UV radiation may cause its degradation. Such applications include perovskite solar cells assembled directly on transparent wood substrates [30], anisotropic transparent paper with high efficiency as a light management coating layer for GaAs solar cell [10], smart photo-responsive windows with energy storage capabilities [31], radiative cooling structural materials [32], smart and energy-saving buildings applications [9,33], and structural elements in architectural construction. [34,35] In addition, due to the worldwide pandemic caused by COVID-19, the use of UV-C radiation for sanitation of surfaces and internal spaces has risen dramatically.

Based on the arguments described above, it is necessary to understand the influence of UV radiation on this type of material that has huge potential applications in the future. According to a review made by [36], there are questions which should be addressed in the future studies to allow industrialization of the technology, such as optical and mechanical stability and the desirability of increased cellulose content. The increased cellulose content was addressed by [29].

A study, conducted by [37], evaluated colour, chemical and optical (transmittance) changes of a transparent wood composite made from poplar wood and epoxy resin with a UV absorber when exposed to UV-A (340 nm) light. To the best of our knowledge, to this date, it is the only study dealing with this issue. Therefore, the aim of this study is to further examine the effect of UV radiation (UV-C, 250 nm) on optical (transmittance and colorimetry) and mechanical stability (hardness) of lignin retaining transparent wood obtained by lignin chromophores deactivation.

## 2. Materials and Methods

### 2.1. Materials and Chemicals

Radially cut basswood (*Tilia*) was purchased from JAF Holz Slovakia s. r. o. (density: 0.53–0.56 g cm^−3^). Sodium silicate, sodium hydroxide, magnesium sulfate, DTPA and H_2_O_2_ (35%), propanol, acetone were purchased from CentralChem s.r.o. Deionised water was prepared directly in the laboratory. MMA and 2,2′-azobis(2-methylpropionitrile) were purchased from Sigma-Aldrich and 2-hydroxyethyl-methacylate with activator were purchased from Epoxy s.r.o.

### 2.2. Lignin Modification

The lignin modification procedure was originally proposed by [29]. Basswood samples with dimensions of 100 × 50 × 1.2 mm (±0.1 mm) were submerged into a lignin modifying solution at 70 °C until the wood became white. The solution was prepared by mixing chemicals in the following order: deionized water, sodium silicate (3.0 wt%), sodium hydroxide solution (3.0 wt%), magnesium sulphate (0.1 wt%), DTPA (0.1 wt%), and then H_2_O_2_ (4.0 wt%). Gradually, H_2_O_2_ (35% vol) was added to the solution until the samples became completely white. The samples were then washed with hot deionized water to remove traces of residual chemicals. Finally, the samples were dehydrated with propanol and acetone, subsequently, and stored until polymer infiltration.

### 2.3. Transparent Wood Preparation

Before polymer infiltration, wood samples were dehydrated with ethanol and acetone sequentially. Each solvent-exchange step was repeated three times. MMA was pre-polymerized before infiltration to remove the dissolved oxygen. Pre-polymerization was carried out at 75 °C for 15 min with 0.3 wt% 2,2′-azobis(2-methylpropionitrile) as initiator and the solution was then cooled to room temperature. Subsequently, the bleached wood template was fully vacuum-infiltrated in a pre-polymerized PMMA solution. Finally, the infiltrated wood was sandwiched between two glass slides, packaged in aluminium foil, and the polymerization was performed in an oven at 75 °C for 4 h. Infiltration of 2-hydroxyethyl-methacylate was carried out without pre-polymerization. Activator (0.2 wt%) was mixed with 2-hydroxyethyl-methacrylate and it was allowed to dissolve for 1 h. After full vacuum-infiltration the samples were sandwiched between two glass slides, packaged in aluminium foil, and the polymerization was performed in an oven at 90 °C for 4 h. In total 20 pieces of samples were prepared (10 for each methacrylate). After fabrication of the samples, the resulting product can be considered to be around 40% renewable.

### 2.4. Characterization

**Figure 1:**
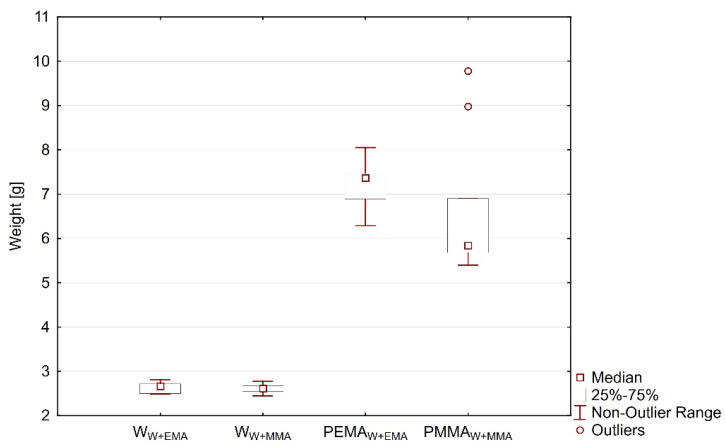
Boxplot of wood and acrylate polymer weight in the samples.

According to the study by Li et al. [29], the transparent wood samples retained up to 80 wt% of lignin leading to a stronger wood template compared to the de-lignified alternative. In this study, the weight loss of the wood component due to the modification of lignin was 21.9 ± 0.9%. After polymer infiltration, a high-lignin content transparent wood with a transmittance of 83%, a haze of 75%, a thermal conductivity of 0.23 Wm K^−1^, and work-to fracture of 1.2 MJ m^−3^ (a magnitude higher than glass) was obtained (MMA samples). Samples prepared for this study did not reach the values of previously mentioned research because of the use of different (more dense) wood. Figure 1 shows a boxplot of wood and acrylate polymer weight in the samples. The average proportion of wood component in the W+MMA and W+EMA samples was 27% and 29%, respectively.

The colorimetry of the samples was performed using by a Colorimeter NR200 Precision (Threenh Technology Co., Ltd.; Shenzhen, China) with the following characterizations: Measuring aperture Φ8 mm, Colour space CIE L*a*b* and Light Source D65. The colour change caused by UV radiation was monitored using the CIE L*a*b* colour space coordinates. In this way, the colour of the measured surface is expressed using three coordinates:

- L*—coordinate on the axis indicating lightness
- a*—coordinate on the axis between red and green
- b*—coordinate on the axis between yellow and blue

To describe the total shift in this colour space, the total colour difference is used, which can be expressed as follows:

(1) $$dE_{t}=\sqrt{{\left(L_{t}^{*}-L_{0}^{*}\right)}^{2}+{\left(a_{t}^{*}-a_{0}^{*}\right)}^{2}+{\left(b_{t}^{*}-b_{0}^{*}\right)}^{2}}$$

where *dE_t_* is the total colour difference at time *t*, $L_{t}^{*}$ is the value of L* at time *t*, $L_{0}^{*}$ is the value of L* before exposure to UV radiation, $a_{t}^{*}$ is the value of a* at time *t*, $a_{0}^{*}$ is the value a* before exposure to UV radiation, $b_{t}^{*}$ is the value of b* at time *t*, $b_{0}^{*}$ is the value of b* before exposure to UV radiation.

The transmittance was measured using a modified photometer RMG2.1 (Heil Metalle GmbH, Mülheim, a. d. Ruhr, Germany). The measuring area was 20 mm × 20 mm.

For the measurement of the hardness Digital Shore D Hardness Tester—Sauter HD (Sauter GmbH, Balingen, Germany) was used.

The UV ageing (an accelerated weathering test) has been carried out in a UV chamber. The samples were irradiated for 35 days. All measurements have been done after 7 days of UV exposition. The ageing has been done under a temperature of 50 °C. As a source of UV-C radiation, 4 germicidal fluorescent lamps Philips TUV 15 W (Piła, Poland) were used. The efficiency of the fluorescent lamp was 32%. UV-C radiation (wavelength 250 nm) reached a power output of 4.9 W and the volume of the chamber was 50 L. The samples were placed 100 mm from the UV lamps in every direction. The irradiance flux density was 16.07 W m^−2^ and the inner surface of the chamber was made of stainless steel with a 50% reflectance factor.

For FTIR analysis, Varian FT-IR Spectrometer 660 (Agilent Technologies, Inc., Santa Clara, CA, USA) samples were directly applied to a diamond crystal of ATR GladiATR (PIKE Technology Inc., Madison, WI, USA) and the resulting spectra were corrected for background air absorbance. The spectra were recorded using a Varian Resolutions Pro and samples were measured in the region 4000–400 cm^−1^; each spectrum was measured 146 times, at resolution 4.

All of the measurements were carried out using Stat Soft STATISTICA 10 (StatSoft s.r.o., Praha, Czechia) software. The impact of the exposure time of UV radiation on the total colour difference, transmittance and hardness were evaluated by the Duncan’s test.

## 3. Results

### 3.1. Colorimetry

**Figure 2:**
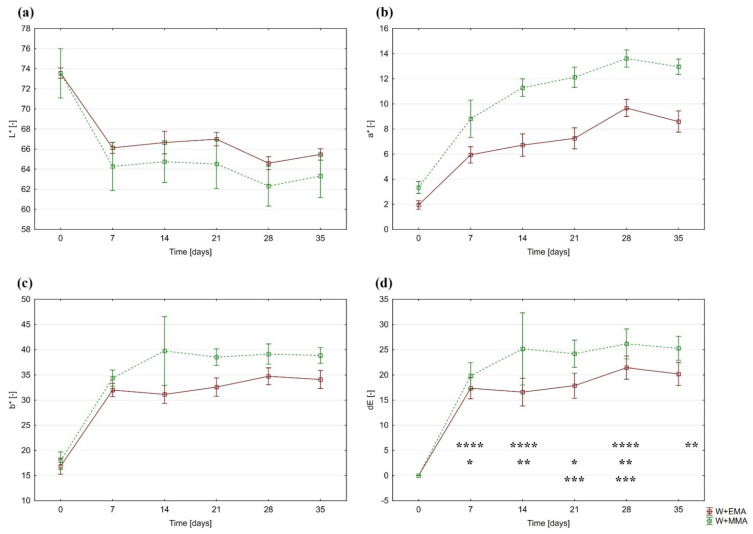
Time dependence of colour components in CIE L*a*b* space and total colour difference: (**a**) Lightness; (**b**) the coordinate a*; (**c**) b* coordinate; (**d**) total colour difference *, **, *** denoted exposure times of W+EMA and **** denoted exposure times of W+MMA at which the total colour differences were statistically significant based on the results of Duncan’s test (difference between 0 days of exposure and other exposure times is obvious without statistical test).

In terms of colour change due to UV radiation, the most significant changes occurred during the first 7 days. This fact is clearly visible in Figure 2. A significant change was observed in all three coordinates, which was subsequently reflected in the value of the overall colour difference.

The average lightness of both measured materials was approximately 73.5. However, after the first 7 days of UV exposure, it decreased to values of around 66 for W+EMA samples and to an average of 64 for W+MMA samples, which represents a reduction of 10% and 17%, respectively.

The a* coordinate also changed most rapidly at the onset of UV exposure. In 7 days, its average value increased from 2 to almost 6 (W+EMA) and from 3.4 to 9 (W+MMA). A less pronounced increase subsequently continued until day 28 of the test. Subsequently, there was a very slight decrease. As with L*, W+EMA samples proved to be less susceptible to changes due to UV radiation.

In the case of the b* values, it is possible to see a similar course as in the case of a*, but after the initial significant increase it changes only slightly over a period of more than 7 days. Although the b* of both materials is very similar in the samples before exposure to UV radiation, the samples of W+MMA acquire higher values than W+EMA due to its influence.

The results of the overall colour difference reflect the changes described in the individual coordinates. A significant colour change occurs mainly during the first 7 days, followed by only a slight increase.

**Figure 3:**
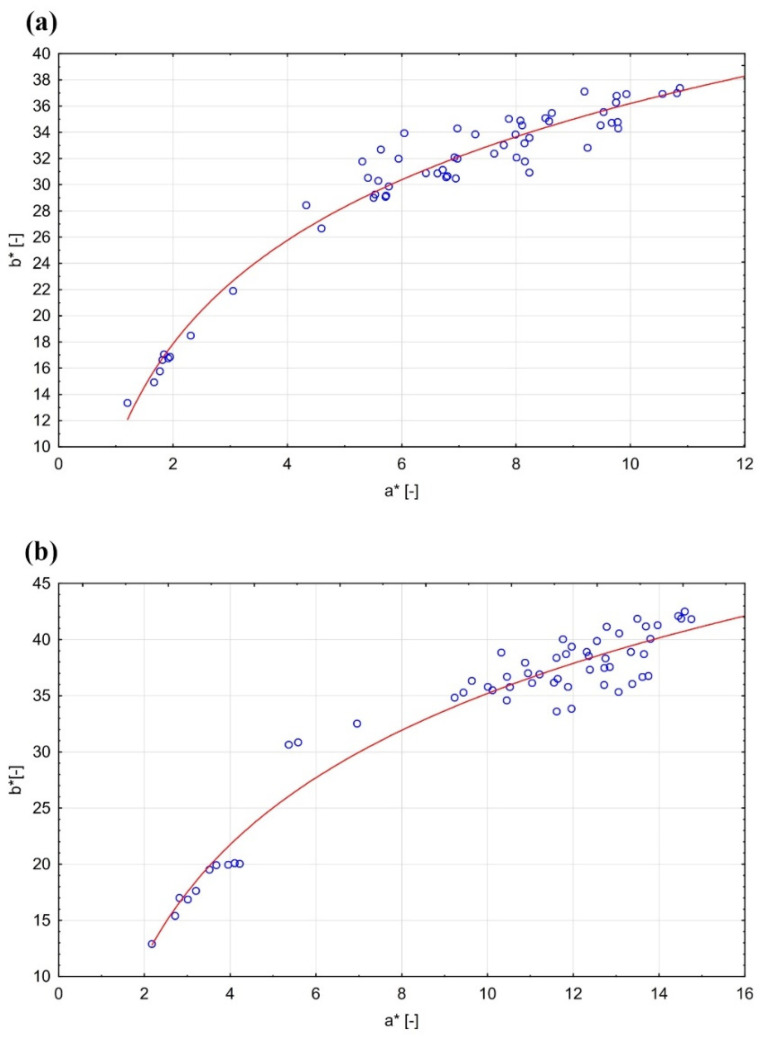
Interdependence of colour space coordinates a* and b*: (**a**) W+MMA; (**b**) W+EMA.

As already mentioned, the graphs corresponding to the values of a* and b* have a similar course. The following equations can be determined from the graphical representation of the measured values (Figure 3):

(2) $$b_{W+EMA}^{*}=9.96+26.24\times \log a_{W+EMA}^{*}$$

(3) $$b_{W+MMA}^{*}=1.83+33.91\times \log a_{W+MMA}^{*}\;\;$$

where $b_{W+EMA}^{*}$ is the coordinate b* for the sample W+EMA, $a_{W+EMA}^{*}$ is the coordinate a* for the sample W+EMA,$\;b_{W+MMA}^{*}$ is the coordinate b* for the sample W+MMA and $a_{W+MMA}^{*}$ is the coordinate a* for the sample W+MMA. The coefficients of determination in these cases are 0.9376 (W+EMA) and 0.8191 (W+MMA).

**Figure 4:**
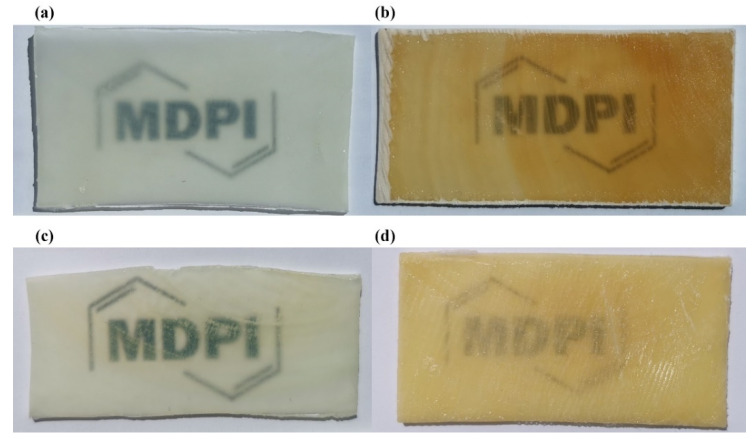
Change of transmittance and colour: W+EMA (**a**) before UV-C irradiation; (**b**) after 35 days of UV-C irradiation; W+MMA (**c**) before UV-C irradiation; (**d**) after 35 days of UV-C irradiation (Source of logo: https://www.mdpi.com/journal/polymers, accessed on 28 May 2021).

The comparison of colour changes in transparent wood infiltrated by 2-hydroxyethyl-methacrylate (a,b) and methyl methacrylate (c,d) before and after 840 h of UV-C irradiation is shown in Figure 4a,b, and in Figure 4c,d. The significant colour darkening (photo yellowing) was observed within the first few hours of exposure, which increases with further exposure.

### 3.2. Transmittance

**Figure 5:**
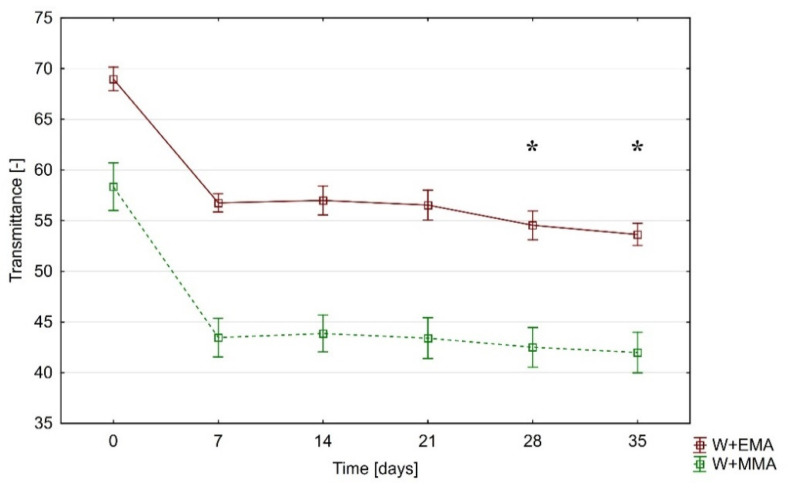
Change of transmittance during the experiment * denoted exposure time of W+EMA at which the total colour differences was statistically significant (in interval from 7 to 35 days) based on results of Duncan’s test (difference between 0 days of exposure and other exposure times is obvious without statistical test).

Depending on the acrylic polymer used, the transmittance values differ significantly, even for samples not exposed to UV-C radiation (Figure 5). While W+EMA transmits almost 69% of light, W+MMA is about 58%. After 7 days, these values decrease to 57% resp. 43% and consequently their change is almost negligible. Throughout the experiment, the W+EMA samples remained significantly more transparent, with the difference between them highlighted by the action of UV-C radiation, as is shown in Figure 5.

### 3.3. FTIR Analysis

**Figure 6:**
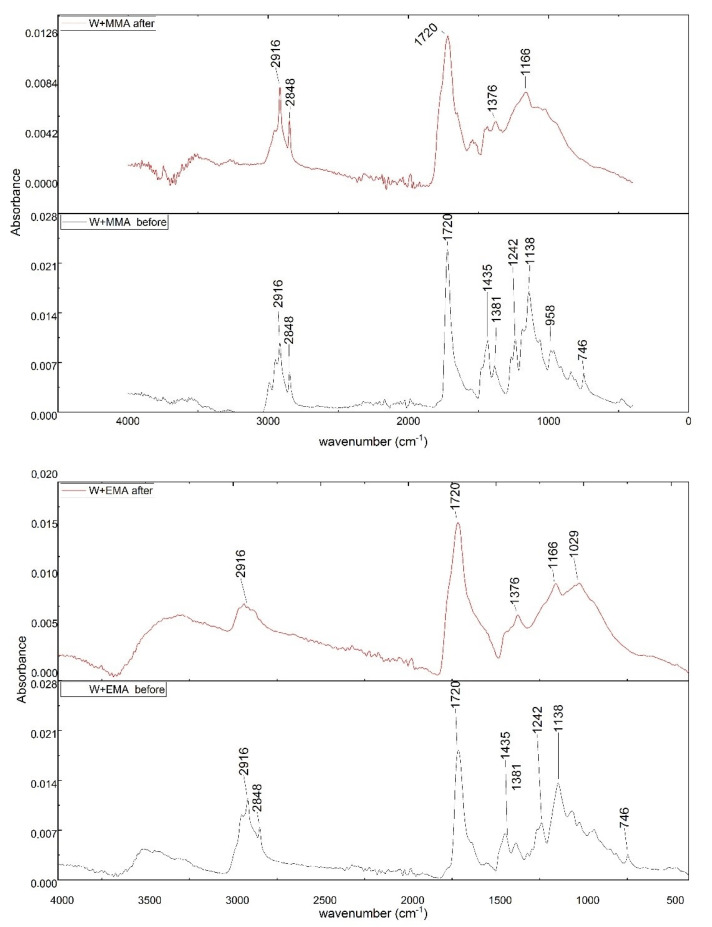
Changes to the chemical structure of W+EMA and W+MMA samples before and after the UV-C irradiation.

Changes to the chemical structure (bond scission/forming) of W+EMA and W+MMA sample after UV-C irradiation are displayed in Figure 6 as infrared spectrum. There are two characteristic bands attributed to the stretching C–O and CH_3_–O of methyl ester, peak at wavenumber 2916 cm^−1^ and 2848 cm^−1^ the –CH stretching aliphatic band of the ethylene segment. It is seen, a very strong peak is visible at 1720 cm^−1^ due to carbonyl (–C=O) stretching vibration of the acrylate ester group, in both samples. Two peaks at 1435 and 1381 cm^−1^ can be attributed to CH_3_ symmetric and asymmetric deformation. At wavenumber 958 cm^−1^ can be seen C–O–C stretching vibration and at 746 cm^−1^ is band characteristic for C–H stretching [38,39,40,41]. However, after irradiation by UV-C, major changes were observed evidencing chemical changes in the polymer samples. The bands that undergo prominent changes are the functionalities of hydroxyl O–H, carbonyl C=O and ester (C–O–C) in region of wavenumber from 746 to 1435 cm^−1^. Other photo products, e.g., carbonyl groups or double bonds may be weakened from the surfaces, leading to reduced absorption [42].

### 3.4. Hardness

**Figure 7:**
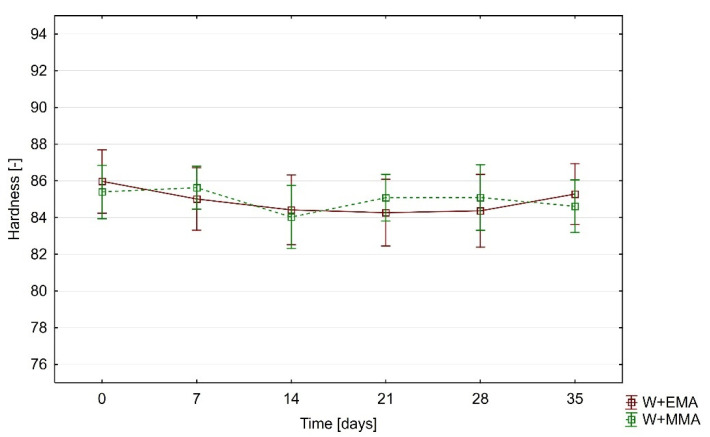
Change of hardness during the experiment (the Duncan’s test proved no statistically significant differences between hardness at all investigated times for both investigated samples).

The hardness of both types of materials was at a very similar level. Its values were initially around 86. In contrast to the optical properties, the hardness of the measured samples is significantly less affected by the UV-C radiation to which the transparent wood samples were exposed. Figure 7 shows its course as a function of the time of UV-C treatment. The hardness of both types of samples was practically the same during the experiment and no differences are apparent between them.

## 4. Discussion

Shore A hardness of PEMA is according to Hourston, Satgurunathan and Varma 78 [43]. A value higher than 95 can be deducted from the graph in the work of Hourston and Schäfer [44]. The W+EMA samples had a hardness of 86 ± 2.3 and were therefore among the data of the mentioned authors.

The hardness of PMMA in the Shore D scale can be found in a higher number of works than in the case of PEMA. According to Poomali, Suresha and Lee, its value is 90 ± 1 [45]. Seeger et al. state a value of 87.5 ± 0.4 [46] and Akinci, Sen and Sen 79 [47]. The data measured in this study for W+PMMA are 85.4 ± 1.9, which is in agreement with the reported values. Because of these similarities as well as the high proportion of acrylic polymer in the samples (approximately 35%), it can be stated that the hardness is much more significantly influenced by the properties of the resin as a component of delignified wood. It was also observed that the samples were more brittle during hardness testing. No cracks and fractures were observed directly after UV-C irradiation. This behaviour was confirmed by the study of [48] where ultraviolet radiation altered PMMA stiffness, resulting in changes in tensile properties, such as reduction in elongation at break and tensile strength.

Light exposure is a major cause of wood degradation, leading to colour change and loss in mechanical properties [49,50,51]. Significant changes were observed after 4 h of irradiation. All *m/z* signals of lignin were either absent or their intensity was considerably reduced, suggesting that lignin underwent an extensive degradation. The irradiation promoted a reduction in the transparency, due to the yellowing [47].

UV degradation of poly(methyl methacrylate) and its vinyltriethoxysilane containing copolymers, was tested using a mercury lamp with a wavelength of 259 nm, situated 10 cm away from the samples and found out that UV irradiation causes changes in the mechanical properties of PMMA [52].

Wochnowskia et al. [53] irradiated PMMA by UV-laser light with different wavelengths (193 nm, 248 nm and 308 nm) in order to investigate the photolytic degradation of the physico-chemical molecular structure and reported that, during the UV-irradiation (248 nm), there was the existence of methyl formate, a great amount of methanate, methanol and additionally the occurrence of methyl and other molecule fragments of the polymer side-chain even at a low irradiation dose. At this irradiation dose, side chain cleavage from the polymer main chain takes place yielding mechanical densification of the polymeric material due to Van-der-Waals forces with a subsequent increase in the refractive index.

From the above-mentioned arguments we can conclude that the change of favourable optical properties of transparent wood (transmittance and colour) was caused by the degradation of both components, the acrylic polymer as well as the wood itself.

## 5. Conclusions

Transparent wood, combining many advantageous properties, is an emerging new material for light-transmitting and environmentally friendly applications. There is an increasing number of research teams who introduce new methods of fabrication and new ways to use transparent wood. Therefore, it is crucial to know how this material behaves under various conditions.

Exposure to UV-C sources has a significant effect on the colour of transparent wood. It was mostly pronounced from the beginning of the test (during the first 7 days). Samples became darker with increasing exposure time and their colour shifts towards shades of red and yellow which can be possibly explained by the reactivation of chromophores. The values of the coordinates a* and b* show an interdependence that appears to be logarithmic. W+MMA samples are more prone to discolouration due to UV-C radiation than W+EMA samples.

The transmittance of light through the measured samples of transparent wood was significantly affected by the action of UV-C radiation. As in the case of colour changes, the UV-C effect was most pronounced at the beginning and had only a minimal effect in the later stages. W+EMA had higher light transmission and its reduction due to UV-C was less pronounced than in the case of W+MMA.

The influence of UV-C on shore D hardness of W+EMA and W+MMA is significantly lower than in the case of optical properties. The differences between these materials are not statistically significant. The measured values show that the resulting hardness of transparent wood depends mainly on the hardness of the acrylic polymer used.

In a previously mentioned study, the impact of UV-B radiation on the optical and mechanical properties of transparent wood has been investigated. The UV-B radiation was used for ageing acceleration. Due to the SARS-CoV-2 virus pandemic, the UV-C radiation (for virus deactivation purpose) began to be used massively. However, there were no data concerning the impact of UV-C radiation on transparent wood key properties before this study. This is the first study revealing the impact of UV-C radiation on key optical and mechanical parameters of transparent wood. Obtained results also proved that UV-C radiation (at irradiance flux of 16 W·m^−2^ during 35 days) has virtually no effect on the transparent wood (W+EMA and W+MMA) shore D hardness. Obtained results also proven that the impact of UV-C radiation on the optical characteristics of transparent wood (at stated irradiance flux) is significant only for the first 7 days (in the following days the impact was only negligible).

In future research, it is necessary to evaluate the effect of different wavelengths on the properties of transparent wood and also to describe the time period during which the highest degradation occurs.

## Data Availability

Not applicable.
